# Michigan State Veterinary Medical Association

**Published:** 1896-03

**Authors:** William Jopling

**Affiliations:** Secretary


Secretary.

### MICHIGAN STATE VETERINARY MEDICAL ASSOCIATION.

THE fourteenth annual meeting was held in the parlors of New Grand Hotel, Lansing, Mich., February 4 and 5, 1896. President George C. Moody, of Mason, called the meeting to order at 7.30 P.M., Tuesday, February 4th. Roll-call showed the following members present, viz.: Drs. J. Hawkins, D. G. Sutherland, D. Cummings, J. A. Dell, S. Brenton, E. A. A. Grange, J. W. Ferguson, C. W. Stowe, B. C. McBeth, J. C. Whitney, William Jopling, George C. Moody, W. W. Thorburn, G. W. Dunphy, T. G. Duff, G. H. Carter, W. A. Giffen, A. Campbell, R. H. Kestell, James Drury, W. M. Burdick, James Harrison, H. M. Gohn, W. A. McLean, J. D. Rutherford, Judson Black, and Wallace McQueen. Drs. A. McKercher, H. F. Palmer, F. M. Blatchford, George E. Metcalf, and J. B. Stevens were present as visitors.

President Dr. George C. Moody delivered the annual address of welcome. After thanking the Association for the honor conferred upon him in electing him President and expressing a hope that the meeting might prove a satisfaory one, he went on to say to the members that it was not his intention to tire them with a long address, as he thought the time could be more profitably occupied in discussion of questions which will be more beneficial to us in our future practice and would also have a tendency to make our meetings more interesting, and that as we had a very good programme to work from, there was no doubt but what we would have a good opportunity to exchange ideas in the discussion of papers, and the several reports would no doubt make the meeting one of interest and zeal. He said there were a great many advantages to be gained by attending these meetings, and that it should be a continued effort on the part of every member to make our meetings a success. He said this might be accomplished by members keeping records of cases that are of peculiar nature, with the treatment thereof and of the results obtained, and reporting them at our meetings, which will be of great importance to us in the way of bringing forth discussions and the knowledge of new remedies and appliances, which will serve to make us more proficient in their use and at the same time advance us in the practice of our profession. It also gives us a chance to meet and become acquainted with our brother-practitioners as well. Herein lies the secret of having good attendance, the elevating of the profession, and a desire on the part of practitioners to become members of the Association. It will also be the means to assist in the abolishing of empiricism and quackery.

One thing more I will mention as an adjunct to keep up the interest in our profession, that is, the veterinary journals. They are something that should be found in the office of every veterinarian; by them you will not only be able to keep pace with what is transpiring in veterinary science at home, but may learn much of the doings and progress of the profession from across the ocean, in our sister countries. As there are a number of these journals edited and the subscription price within the reach of all, he most earnestly recommended the taking of some one of them if not already subscribers. In this connection the President gave the following quotation: "He who does not fertilize his mind with the current literature of the day will soon become barren. A man cannot become mentally great by his own thoughts and information."

He also spoke at some length on the question of legislation, being heartily in favor of it; he said that if every one would put his shoulder to the wheel there was no doubt in his mind that we could secure the passage of a good liberal Act. After calling attention to an amendment to our code of ethics to be voted on at this meeting, he concluded, by saying: "To the Secretary, Dr. Jopling, too many thanks cannot be given for the excellent work he has done this year, like years past, in the interest of the Association."

It was moved and supported that a vote of thanks be given President Moody for his excellent address and that the same be spread upon the minutes; carried.

Minutes of previous meeting were read and approved. The following gentlemen made application for membership: Dr. A. McKercher, Lansing-Ontario Veterinary College, year 1893. Vouchers, Drs. James Drury, V.S.; H. M. Gohn, V.S. Dr. H. F. Palmer, D.V.S., Napoleon, Veterinary Department, Detroit College of Medicine, 1895. Vouchers, C. M. Higginson, D.V.S.; J. D. Rutherford, M.D.V.S. Dr. F. M. Blatchford, V.S., Brighton, Ontario Veterinary College, year 1893. Vouchers, H. M. Gohn, V.S.; William Jopling, V.S. Dr. George E. Metcalf, D.V.S., Detroit, Detroit College, year 1893. Vouchers, J. D. Rutherford, M.D.V.S.; S. Brenton, V.S. Dr. R. H. Alexander, V.S., Leslie, Ontario Veterinary College, year 1892. Vouchers, George C. Moody, V.S.; H. M. Gohn, V.S. Dr. J. B. Stevens, V.S., Yale, Ontario Veterinary College, year 1891. Vouchers, W. M. Burdich, V.S.; William Jopling, V.S. The applications were referred to the Executive Committee, who reported favorably on them all. The report was accepted and adopted, and the Secretary instructed to cast the ballot of the Association for them collectively. Ballot was so cast, and the six applicants declared duly elected to membership.

Secretary's report was read and ordered filed and a vote of thanks extended.

The Treasurer's report was read and referred to the Finance Committee.

At this time a recess was taken for payment of fees and dues. When called to order again, Dr. Drury reported for the Finance Committee that they found everything correct in connection with Treasurer's books.

Report of Finance Committee was accepted and ordered filed.

Prof. J. D. Rutherford, of Veterinary Department, Detroit College of Medicine, made a very able report for the Committee on Intelligence and Education. The report was accepted and ordered placed on file.

Prof. E. A. A. Grange, of the Agricultural College, Lansing, and State Veterinarian, reported for the Committee on Diseases. He said epizoötics were rare in the State for past year. In an official capacity he had been called to investigate twenty cases of glanders and ten cases of a variety of troubles, such as hog cholera and certain skin disorders.

Drs. J. Hawkins and J. A. Dell, members of the Committee on Diseases, also made short reports, in which paralysis, parturient apoplexy, tetanus, azoturia, and other diseases were discussed.

At this time Professor Grange introduced Mr. H. H. Hinds, President of the Live-stock Sanitary Commission, who made a few appropriate remarks and expressed his pleasure at being present.

Communications were read from Dr. E. W. Wells, Grand Rapids; Dr. H. C. Waun, Claireview; Dr. H. W. Rupright, Sturges; and Dr. W. Horace Hoskins, Philadelphia, Pa., President of the U. S. V. M. A. The same were, upon motion, received and ordered placed on file.

The meeting adjourned at 11.30 P.M. to meet at Dr. W. W. Thorburn's infirmary at 9 A.M., to witness the operations of laryngotomy, ovariotomy, and castration of a cryptorchid horse.

9 A.M., February 5th, all met at Dr. Thorburn's. The first operator was Dr. W. A. McLean, of Greenville, who operated on a cryptorchid horse. The doctor proved himself a very cool and expert surgeon in that line of work. The operation was completed very rapidly, was a complete success, and favorable comments were heard on all sides.

President Moody removed a fibroid tumor from a dog's eye, and performed ovariotomy on a bitch. The operations were done with neatness and dispatch, and added greatly to the entertainment of those present.

The subject upon which the operation of laryngotomy was to be demonstrated not being ready, it was decided to postpone the operation until 1 o'clock P.M., and in the meantime return to the hotel and hold a forenoon session, which was done, the President calling the meeting to order at 10.30. After roll-call the Committee on Legislation were asked for their report. The report consisted in the Chairman stating that the Committee had done nothing.

The communication from Dr. W. H. Hoskins, read at previous session, was ordered taken from table and re-read. The request contained therein, asking the Association to indorse the Army Bill and its actions, did not meet with favor for the reason that it appeared to discriminate in favor of graduates of United States colleges. If the words, of the United States, in Sec. 3, lines 3 and 4, were stricken out, the Society would have given its most hearty indorsement. It was, therefore, moved, supported, and carried that the Association do not indorse the bill in its present form.

The Secretary read a list of names of delinquent members, which was referred to the Executive Committee with orders to report at afternoon session.

Upon motion, the meeting adjourned for dinner and to meet at Dr. Thorburn's infirmary at 1 o'clock P.M., to witness the operation by Dr. S. Brenton, of Detroit, on a "roarer." At the appointed time all were present. The patient was cast, chloroform administered; when thoroughly under influence of the anæsthetic the patient was laid on its back with feet suspended toward the ceiling. All being in readiness, the operation was proceeded with, an incision about four inches in length being made through the integument, muscles, and trachea, exposing the larynx to view. The effect of the atrophied muscles on the left side of the larynx was beautifully demonstrated in this case. While the arytenoid cartilage on the left side remained stationary and immovable, the one on right side responded regularly to the muscular action during inspiration and expiration. It took some time for the large class present to pass around by the operator and view the larynx one by one, but it was a sight worth seeing and possessed great interest for those present. The collapsed cartilage was then excised and the parts again examined by all present, after which we returned to the hotel and were called to order at 2.30. It being necessary for Dr. W. A. McLean to go home at this time, a vote of thanks was tendered him for his part in entertaining the members.

Dr. J. A. Dell read an interesting and carefully compiled paper entitled, " Some Observations on Conception and Pregnancy in the Mare." Dr. J. Black, of Richmond, read a communication describing a peculiar case occurring in his practice under the head, " Foreign Matter in Alimentary Canal." Prof. E. A. A. Grange entertained the Society with an " Extemporaneous Talk on Tuberculosis and Other Things." Dr. R. H. Kestell, of Ypsilanti, explained a mode of treatment for contracted feet which he recommended very highly. He termed it " Walling the Feet," and applied it in chronic laminitis and navicular arthritis. Professor Grange again took up the subject tuberculosis, and, besides reading a paper on this important question of the day, talked very entertainingly of his experience with the disease in its various phases. President Dr. G. C. Moody read a paper on " Wry Tail." Dr. C. M. Higginson, of Jackson, who was to read a paper entitled " The Veterinary Surgeon," sent telegram that he was unavoidably detained at home. Spirited discussions followed the reading of each paper, but discussion was necessarily limited for want of time.

The following officers were elected: President, Dr. W. W. Thorburn Lansing; First Vice-President, Dr. A. Campbell, Jackson; Second Vice-President, Dr. J. D. Rutherford, Detroit; Third Vice-President, Dr. Judson Black, Richmond; Secretary and Treasurer, Dr. William Jopling, Owosso. Directors: Dr. C. W. Stowe, Saginaw; Dr. B. C. McBeth, Battle Creek; Dr. J. A. Dell, Ann Arbor; Dr. H. F. Palmer, Napoleon; Dr. James Drury, Lansing; Dr. D. Cummings, Port Huron.

It was moved and supported that we have a full two days' meeting next year; carried.

A hearty vote of thanks was given our host and hostess for the kind treatment we had received from them. A vote of thanks was given Dr. S. Brenton, of Detroit, who added so much to the entertainment of the Association.

The newly elected President appointed the following committees: " Intelligence and Education," Drs. Hawkins, Cummings, and Gohn; " Diseases," Drs. Grange, Drury, and Dell; " Finance," Drs. G. W. Dunphy, W. McQueen, and Harrison; " Legislation," Drs. Rutherford, Giffen, and Moody.

Adjournment was then taken and all returned home, well pleased that they had attended the meeting which had proved to be the best ever held by this Society.

WILLIAM JOPLING,
Secretary.

